# The relationship between body mass index and cerebrospinal fluid pressure in children with pseudotumor cerebri

**DOI:** 10.1186/s13052-024-01722-w

**Published:** 2024-08-17

**Authors:** Yakup Çağ, Safiye Güneş Sağer, Merve Akçay, İsmail Kaytan, Elif Söbü, Aydan Erdem, Yasemin Akın

**Affiliations:** 1 Department of Pediatrics, University of Health Sciences, Kartal Dr. Lütfi Kırdar City Hospital, Istanbul, Türkiye; 2 Department of Pediatric Neurology, University of Health Sciences, Kartal Dr. Lütfi Kırdar City Hospital, Istanbul, Türkiye; 3 Department of Pediatric Endocrinology, University of Health Sciences, Kartal Dr. Lütfi Kırdar City Hospital, Istanbul, Türkiye

**Keywords:** Pseudotumor Cerebri, Obesity, Body Mass Index, Cerebrospinal Fluid Pressure, Children

## Abstract

**Background:**

Childhood obesity has become a major global health problem. Obesity is associated with major health problems, such as diabetes, hypertension, dyslipidemia, cardiovascular disease. Obesity is also considered a risk factor for Pseudotumor cerebri (PTC). The present study aimed to investigate the relationship between body mass index (BMI), and cerebrospinal fluid (CSF) pressure in patients with pseudotumor cerebri.

**Methods:**

A total of 48 children diagnosed with PTC, who were aged < 18 years and followed up in the pediatric clinic were included in the retrospective study. National BMI percentile curves were used for reference. We investigated statistically the relationship between BMI, clinical and laboratory results, and CSF pressure in patients.

**Results:**

Of total patients 27 were female (56.25%) and 21 were male (43.75%). With regard to the BMI percentile, 20 (41.67%) were overweight or obese. CSF pressure was higher in overweight and obese patients compared to children with BMI in normal ranges (*p* < 0.05). A statistically significant positive correlation was also observed between BMI and CSF pressure values and between monocyte and CSF values (*p* < 0.05).

**Conclusions:**

The results of the present study indicate a direct relationship between CSF pressure and BMI in children with PTC. Appropriate diet, exercise, and medical treatment in overweight and obese children can make a significant contribution to the treatment of PTC. Additionally, a significant correlation was observed between CSF pressure and monocyte levels.

## Introduction

Pseudotumor cerebri (PTC), also known as idiopathic intracranial hypertension or primary intracranial hypertension, is defined as increased cerebrospinal fluid (CSF) pressure inside the brain without hydrocephalus, masses, or meningeal abnormalities [1]. PTC can be observed in children of all ages and is more prevalent in obese young adult women [2]. The incidence of PTC ranges from 0.7 to 0.9 per 100,000 children each year [3, 4]. Although the pathogenesis is not fully understood, certain associated causes have been suggested, including increased CSF production and obstruction in circulation pathways [5]. Clinically, PTC manifests as headache, visual disturbances, papillary edema, vision loss, and in some cases tinnitus [6]. Symptoms usually tend to improve over weeks to a few months, nevertheless a fulminant course may be seen in certain cases. The most feared outcome is considered permanent loss of vision. Permanent loss of visual acuity or visual field was reported in 19% of cases [7].

Childhood obesity has become a major global health problem. Obesity is associated with major health problems, such as diabetes, hypertension, dyslipidemia, cardiovascular disease, asthma, sleep apnea, psychological, and social stress. Obesity is also considered a risk factor for PTC. Furthermore, it was reported that obesity induced an increase in intraocular pressure [8].

The present study aimed to investigate the relationship between body mass index (BMI), and CSF pressure in patients with pseudotumor cerebri in our clinic.

## Materials and methods

### Study design

A total of 48 children diagnosed with PTC, who were aged < 18 years and followed up in the pediatric clinic of our hospital between January 1, 2021 and August 1, 2022 were included in the retrospective study. Required ethics committee approval was obtained from the ethics committee of Kartal Dr. Lütfi Kırdar City Hospital (Decision No. 2022/514/228/13). The study was performed pursuant to the ethical principles stipulated in the World Medical Association (WMA) Declaration of Helsinki–Ethical Principles for Medical Research Involving Human Subjects. Parental consent was not obtained due to the retrospective design of the study.

Patients with chronic lung, heart, or kidney disease, central nervous system disease, or immunosuppression, as well as those with conditions causing secondary intracranial hypertension, such as primary or secondary aldosteronism, adrenocorticotropic hormone deficiency, diabetes mellitus, secondary adrenal insufficiency, Addison's disease, medication-induced intracranial hypertension, and infectious intracranial hypertension, were excluded from the study.

### PTC diagnosis

The Modified Dandy criteria revised by Friedman in 2014 were used for the diagnosis of pseudotumor cerebri syndrome [9].

### BMI and percentile calculation

BMI was calculated in children as body weight in kilograms divided by height in meters squared and BMI percentile curves were constructed according to gender and age (in months). National BMI percentile curves were used for reference [10]. Percentile values at < 5, 5 to < 85, 85 to 95, and > 95 were considered to indicate underweight, normal weight, overweight, and obesity, respectively. The children included in the study were divided into two groups according to BMI: a normal weight group (5–85 percentile) and an overweight and obese group (> 85 percentile).

### Statistical analysis

All data were recorded and analyzed in electronic environment using the Statistical Package for the Social Sciences (SPSS) for Windows version 22. In the analysis of the data, first, the relevant hypotheses were tested to decide, which tests (parametric/nonparametric) would be applied. The Kolmogorov–Smirnov test and kurtosis and skewness values were investigated to test the normal distribution hypothesis and further analyses were carried out with parametric tests. Independent sample t-test and one-way analysis of variance were used for two independent group comparisons and for more than two groups, respectively. Pearson correlation analysis was used to analyze the relationship between numerical variables. A *p* value of 0.05 was considered statistically significant for the values obtained in the scope of the study.

## Results

Of 48 patients diagnosed with pseudotumor cerebri included in the study, 27 were female (56.25%) and 21 were male (43.75%). The mean age of the patients and mean CSF pressure were 143.47 ± 44.21 (months) and 39.32 ± 13.28 cmH2O, respectively (Table 1). With regard to the BMI percentile, 28 (58.33%) of the children had normal weight and 20 (41.67%) were overweight or obese. A summary of statistical data on other clinical and laboratory results of the patients are given in Table 1.

**Table 1:** Summary statistics on patients' demographic, clinical and laboratory findings

Variable	$\overline{{\varvec{X}} }$±Ss	Med(Min-Maks)	Percentile 25/75
Age (month)	143,50 ± 44,20	150(45–205)	111,50–180,50
Weight (kg)	50,50 ± 21,50	51(19–104)	32–66
Height (cm)	150,10 ± 17,80	155(109–180)	141,50–161,50
BMI Percentile	66,40 ± 32	78,50(7–99)	36–97
CSF pressure (cmH2O)	39,30 ± 13,30	37(21–82)	30,50–45
White blood cells (10^3/uL)	8317 ± 2324	7995(4250–15910)	6590–9380
Hemoglobin (gr/dl)	12,80 ± 1,10	12,70(10–15,4)	12,10–13,40
Lymphocyte (10^^3^/uL)	2867 ± 1162	2575(870–6100)	2145–3445
Neutrophil (10^^3^/uL)	4564 ± 1655	4395(2220–9210)	3240–5615
Neutrophil/Lymphocyte	1,84 ± 1,02	1,63(0,47–6,38)	1,16–2,18
Monocyte (10^^3^/uL)	610,40 ± 174,90	605(250–1120)	500–715
Eosinophil (10^^3^/uL)	210,80 ± 178	185(0–710)	80–325
Basophil (10^^3^/uL)	69,40 ± 125,30	40(0–700)	20–50
Platelet (10^^3^/uL)	297,40 ± 79,10	306(64–459)	245–348,50
MPW (um^3^)	9,81 ± 1,08	9,65(7,2–12,9)	9,20–10,50
Lactate dehydrogenase (U/L)	241 ± 59,40	232(132–376)	201–281
Glucose (mg/dl)	95,90 ± 15,30	92(65–144)	88–101
Aspertate transferase (U/L)	21,60 ± 6,90	21(9–37)	17–26
Alanine transaminase (U/L)	14,30 ± 7	11,50(6–36)	9,50–16,50
C-reactive protein (mg/L)	2,70 ± 4	1,50(0,1–20,3)	0,40–2,98
Creatinine (mg/dl)	0,50 ± 0,10	0,50(0,30–0,90)	0,40–0,60
D-dimer (μg/L)	200 ± 14,10	200(190–210)	190–210

*CSF* Cerebrospinal fluid, *BMI* Body mass index, *MPW* Mean platelet volüme

Of the patients diagnosed with PTC, 31 (64.58%) had headache, 9 (18.75%) had dizziness, and 22 (45.83%) had visual complaints. Papillary edema occurred in 44 patients (91.67%).

There was no statistically significant difference by headache, dizziness, visual problems and papillary edema, and CSF pressure values (*p* > 0.05) (Table 2).

**Table 2:** Relationship between cerebrospinal pressure values and variables

Variables	Group	n	$\overline{{\varvec{X}} }$±Ss	t	p
Headache	Yes	31	38,79 ± 13,49	-0,37	0,71
	No	17	40,29 ± 13,23		
Dizziness	Yes	9	39,39 ± 6,75	0,02	0,99
	No	39	39,31 ± 14,44		
Vision problem	Yes	22	38,45 ± 12,27	-0,41	0,68
	No	26	40,06 ± 14,28		
Papilledema	Yes	44	39,24 ± 13,83	-0,14	0,89
	No	4	40,25 ± 4,27		
BMI Group	Normal	28	34,73 ± 8,22	-3,08	0,01
	Overweight or Obese	20	45,75 ± 16,29		

*t* Independent samples t test, *BMI* Body mass index

There was no statistically significant difference by headache, dizziness, visual problems and papillary edema, and BMI (*p* > 0.05). No statistically significant relationship was found between BMI and CRP (*p* > 0.05).

CSF pressure was higher in overweight and obese patients compared to children with BMI in normal ranges (*p* < 0.05) (Table 2).

**Fig. 1:**
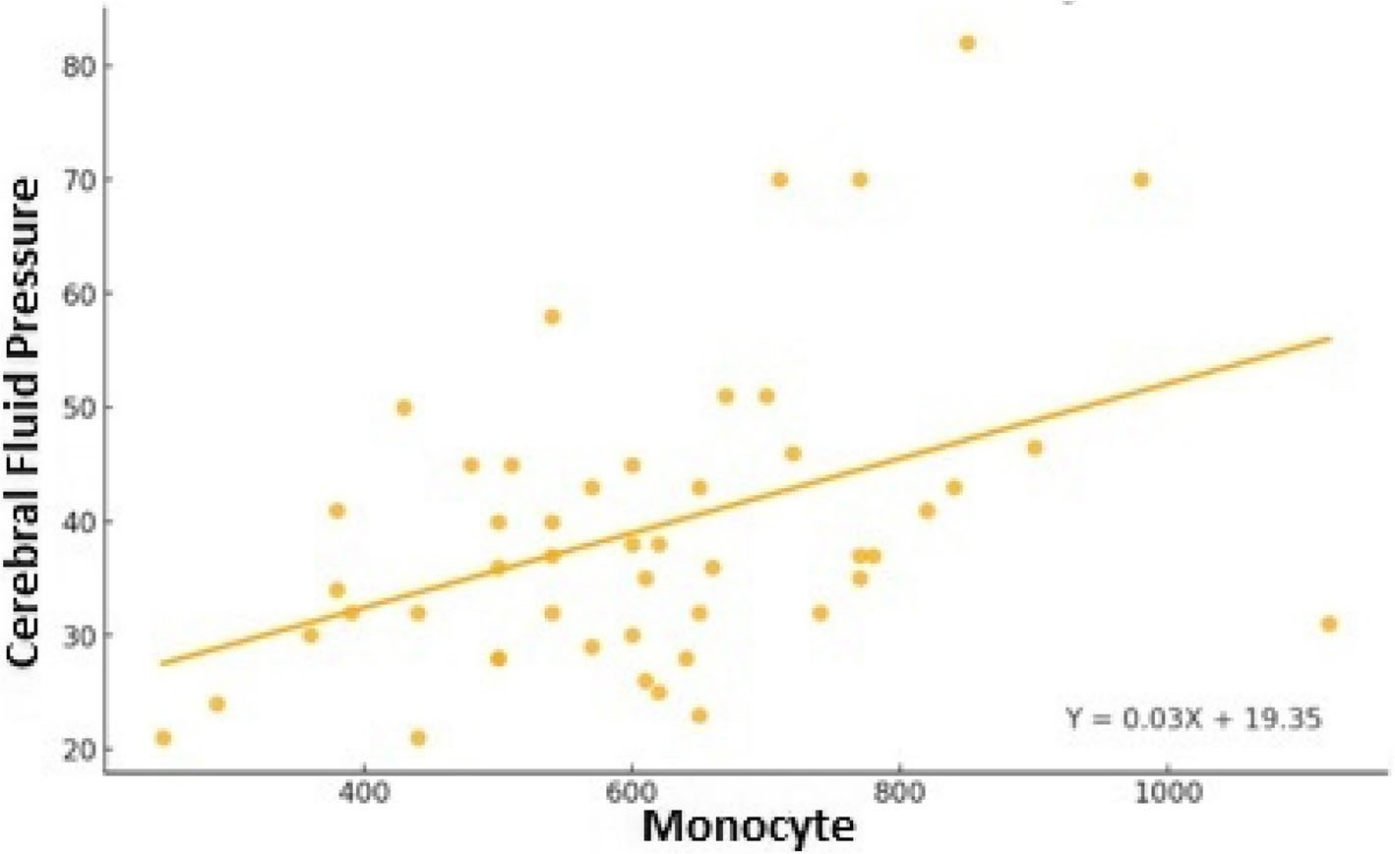
Scatter Plot of Monocyte vs Cerebral Fluid Pressure

**Fig. 2:**
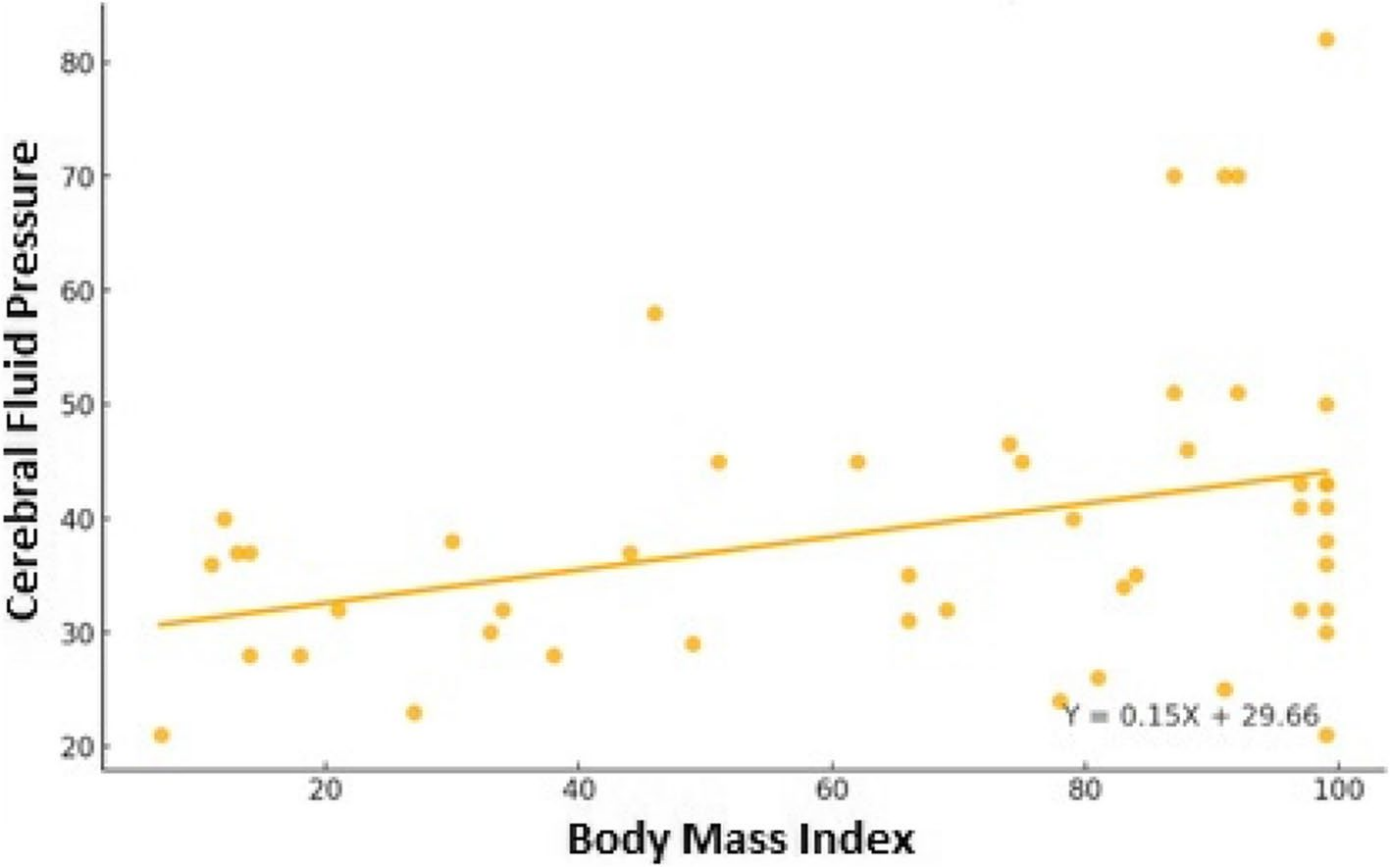
Scatter Plot of Body Mass Index vs Cerebral Fluid Pressure

Laboratory tests showed a statistically significant positive correlation between monocyte and CSF values (r: 0.43; *p* < 0.05) (Table 3), (Fig. 1). A statistically significant positive correlation was also observed between BMI and CSF pressure values (r: 0.35; *p* < 0.05) (Table 3), (Fig. 2).

**Table 3:** Relationship between cerebrospinal pressure values, laboratory findings and BMI

		CSF pressure
Hemoglobin	r	0,10
	p	0,49
Neutrophil	r	0,05
	p	0,76
Lymphocyte	r	-0,08
	p	0,58
Monocyte	r	0,43
	p	**0,00**
Eosinophil	r	0,11
	p	0,45
Basophil	r	-0,17
	p	0,26
MPW	r	0,00
	p	0,98
C-reactive protein	r	-0,09
	p	0,58
BMI	r	0,35
	p	**0,01**

*r* Pearson correlation coefficient, *CSF* Cerebrospinal fluid, *BMI* Body mass index, *MPW* Mean platelet volüme

No significant correlation was observed between hemoglobin, neutrophils, lymphocytes, basophils, eosinophils, neutrophil/lymphocyte ratio, and other parameters measured by CSF pressure (*p* > 0.05) (Table 3).

## Discussion

In the present study, we investigated the relationship between BMI, clinical and laboratory results, and CSF pressure in patients diagnosed with PTC who were followed up in our clinic. In summary, the rate of female sex was higher, 41.67% of the children were overweight or obese according to the BMI percentile. CSF pressure was higher in overweight or obese children compared with children with normal BMI ranges, and there was a statistically significant positive relationship between BMI percentile values and CSF pressure values. A statistically significant positive correlation was observed between monocyte values and CSF pressure values. Previous studies on PTC reported that in general, there was no sex difference in the prepubertal period in childhood, but it was more prevalent in girls after puberty and among adults [1, 11, 12]. In this study, the mean age of our patients coincided with the pubertal period and consistent with the previous studies in the relevant literature, PTC was slightly more prevalent in female sex.

World Health Organization reported that childhood obesity was one of the most critical problems of the twenty-first century. Today, more than 100 million children are obese [13]. Childhood obesity affects almost all organ systems, leading to a number of diseases, such as cardiovascular disease, type 2 diabetes, and various endocrine disorders. Obesity can also contribute to the development of conditions, such as PTC, depression, and cognitive disorders, which may lead to poor academic success [14]. Previous studies reported that obesity was an important risk factor for the PTC development [15–17]. In a study involving 165 children in the United States of America, about half of the patients were overweight (23.40%) or obese (33.10%) [18]. An Italian study conducted in 2020, which included 37 pediatric cases, reported that the vast majority of cases (97%) were overweight or obese [19]. Furthermore, in a study involving adults in the USA, it was reported that 79.2% of idiopathic PTC cases were obese [20]. Upon literature review, it was found that obesity was more prevalent by age in PTC cases [3, 4, 11]. In the present study, about 42% of the cases were obese or overweight. Given the fact that approximately 25% of children in Turkey are overweight or obese [21], our study indicated that obese or overweight children were at increased risk to develop PTC. The potential mechanisms underlying the link between obesity and the development of PTC are not fully known. However, overweight and obesity may contribute to increased intracranial pressure through different mechanisms. It is observed that venous dysfunction is more common in obese patients. Decreased intracranial venous drainage may result in decreased CSF absorption rate and consequently increased intracranial pressure [15]. On the other hand, the increased prevalence of obstructive sleep apnea and high serum and CSF leptin levels in overweight patients may be another causal mechanism [22].

Pseudotumor is more commonly observed in obese. Improving the symptoms of PTC and reducing the risk of complications are crucial. Therefore, we think that weight loss will reduce PTC symptoms in obese and overweight children with PTC. According to magnetic resonance findings, acetazolamide treatment and weight loss have been reported to be effective treatment approaches in children diagnosed with PTC [23]. Calorie-deficit diets and medical weight loss strategies have been shown to improve PTC cases [24].

A study in United Kingdom in 2020 reported a positive correlation between elevated CSF pressure and severity of clinical prognosis [25]. Similarly, a Turkish study reported significant positive correlation between the degree of papillary edema and elevated CSF pressure [26]. It has also been reported that obesity is associated with an increase in intraocular pressure and a decrease in ocular pulse amplitude in children and adolescents, independent of insulin resistance [8]. Another important result of the present study is the direct relation between BMI and elevated CSF pressure. In other words, the higher the BMI, the higher is the CSF pressure. Therefore, we suggest that obesity directly affects the clinical course of PTC and increases its severity. Appropriate diet and exercise in addition to medical treatment in the treatment of PTC in overweight and obese children would make a significant contribution [27].

There was a significant positive correlation in the present study between elevated CSF pressure and monocyte levels. It was reported that monocytes were not generally affected by obesity [28]. Proinflammatory cytokines such as leptin, IL-2, 17, macrophage chemotactic protein-1, plasminogen activator inhibitor-1 and neutrophil-to-lymphocyte ratio are known to be increased in PTC patients [29, 30]. To the best of the authors’ knowledge, there was no study that investigated the relationship between CSF pressure and monocyte values. Although this result shows that monocyte values can be used as a marker for predicting the clinical course of PTC, further studies are needed in this regard.

## Limitation

The main limitation of this study is that it was designed as a single-center study. Nevertheless, the results of this study are important and contribute to the literature.

## Conclusion

In conclusion, obesity and overweight is a significant risk factor for PTC development, and weight loss should be considered as a potential strategy for managing symptoms. However, further clinical studies and long-term follow-up investigations are needed in this regard. These studies could help us better understand the impact of weight loss on PTC symptoms and guide clinical practices. Additionally, a significant correlation was observed between CSF pressure and monocyte levels. However, since there was only a limited data available in the relevant literature, further research may prove to be instrumental in answering the question, “Can monocyte values be used as a marker for predicting the clinical course of PTC?”.

## Data Availability

The datasets used and/or analysed during the current study are available from the corresponding author on reasonable request.

## Abbreviations

PTC: Pseudotumor cerebri
BMI: Body mass index
CSF: Cerebrospinal fluid
